# Loading or Unloading? This Is the Question! A Multi-Season Study in Professional Football Players

**DOI:** 10.3390/sports12060148

**Published:** 2024-05-28

**Authors:** Mauro Mandorino, Antonio Tessitore, Mathieu Lacome

**Affiliations:** 1 Performance and Analytics Department, Parma Calcio 1913, 43121 Parma, Italy; mlacome@parmacalcio1913.com; 2 Department of Movement, Human and Health Sciences, University of Rome “Foro Italico”, 00135 Rome, Italy; antonio.tessitore@uniroma4.it; 3 Sport Expertise and Performance Laboratory, French National Institute of Sports (INSEP), 75012 Paris, France

**Keywords:** football, load monitoring, fatigue, periodization, readiness

## Abstract

This study examined the impact of training load periodization on neuromuscular readiness in elite football players using the Locomotor Efficiency Index (LEI) as a measure of performance optimization. Throughout the 2021/22 and 2022/23 seasons, 106 elite male players (age: 19.5 ± 3.9 years) from an Italian professional football club were monitored using Global Positioning Systems (GPS) external load data. The LEI was derived from a machine learning model, specifically random forest regression, which compared predicted and actual PlayerLoad™ values to evaluate neuromuscular efficiency. Players were categorized by weekly LEI into three readiness states: bad, normal, and good. Analysis focused on the variation in weekly LEI relative to weekly load percentage variation (large decrease, moderate decrease, no variation, moderate increase, large increase), which included total distance, high-speed distance (above 25.2 km/h), and mechanical load, defined as the sum of accelerations and decelerations. Statistical analysis showed significant differences only with variations in total distance and mechanical load. Specifically, reducing weekly loads improved LEI in players in lower readiness states, while maintaining or slightly increasing loads promoted optimal readiness. This approach enables coaches to tailor training prescriptions more effectively, optimizing workload and recovery to sustain player performance throughout a demanding season.

## 1. Introduction

In elite football, the competitive phase (i.e., in-season) typically extends over 9–10 months. During this period, matches are played almost every week, with some weeks featuring up to three matches within a 7 day span [1,2]. Ensuring a team’s sustained peak performance throughout the entire season is paramount, especially in light of potential player fatigue and diminished performance resulting from the weekly demands of competition [3]. Coaches may incorrectly attribute performance declines to players’ fitness deficiencies, potentially making the error of increasing their weekly workload. In fact, according to the fitness–fatigue paradigm [4], performance is considered the result of both fitness and fatigue components, which are both induced during a training session [5]. Hence, a sufficient level of recovery between training sessions and competition is essential to optimize players’ performance, minimize the debilitating effects associated with fatigue, and reduce their risk of injury [6].

Achieving these goals requires a systematic periodization of training processes to ensure players’ optimal physiological adaptations to training demands [7,8]. Then, to quantify the effectiveness of such periodization strategies, specific indicators suitable to identify the general fatigue status of the athletes are needed [6]. These markers could include salivary hormones [9], heart-rate-derived indices [10], psychophysiological indicators [11], and neuromuscular indices [12]. To be considered valid fatigue markers, these should be sensitive to the variations in weekly training load [6,13]. Consequently, numerous researchers have explored the effectiveness of these markers in response to fluctuations in training load. For instance, Thorpe et al. [6] found that during in-season, the perceived ratings of wellness of elite soccer players were sensitive to the fluctuations in their training loads. Similarly, Nobari et al. [14] found a correlation between players’ weekly acute and chronic workloads and their weekly values of fatigue, stress, delayed onset muscle soreness (DOMS), and sleep quality. In another study, Thorpe et al. [15] found that the individual fluctuations in perceived ratings of fatigue were correlated with fluctuations in high-speed running distance covered in the previous two, three, and four days. In particular, the authors observed that the fatigue score worsened by one unit for every additional 400 m of the total high-speed running distance. Therefore, perceived ratings of fatigue showed a correlation with fluctuations in total high-speed running distance recorded in the previous days.

These findings imply that the individual training load cumulated over the previous week could increase players’ fatigue status and impair their neuromuscular readiness. Periodizing training loads is therefore essential to reduce fatigue prior to matches, especially in elite football where players could compete up to three times per week. On that subject, Gastin et al. [16] observed that manipulating weekly training loads altered the subjective ratings of physical and psychological wellness among elite Australian football players, underscoring the benefits of unloading periods for both physical and mental health. Although these markers are sensitive to fluctuations in training load, they have several practical limitations, including subjective assessments (e.g., questionnaires, scales), time-consuming procedures (e.g., heart rate variability evaluation), and invasiveness (e.g., blood markers). To address these limitations, using supervised machine learning techniques (ML), Mandorino et al. [17] developed a new Locomotor Efficiency Index (LEI) to assess players’ neuromuscular status and readiness resulting from their training/match activity. ML offers numerous benefits, particularly in simplifying the daily assessment of neuromuscular readiness and providing a non-intrusive method to detect potential fatigue states, thereby reducing the necessity for athletes to undergo extensive testing. This study’s findings indicated that the LEI varied throughout the season and across different days of the week. Notably, when players faced increased weekly loads, measured by total distance, high-sprint distance (>25.2 km/h), and the number of accelerations (>3.5 m/s^2^), the index significantly decreased. These results underscore the critical role of managing training loads to minimize fatigue and optimize match-day readiness.

Consequently, this study aims to explore how various training load periodization strategies, specifically adjustments to the weekly training load, affect the neuromuscular status of football players, as indicated by the LEI. The research examines various weekly training scenarios within an elite football club: (1) decreasing the weekly training load, (2) maintaining the same weekly training load, and (3) increasing the weekly training load.

## 2. Materials and Methods

### 2.1. Study Design

This observational longitudinal study was conducted over two consecutive football seasons (2021/22 and 2022/23) to analyze locomotor efficiency and training load variations among elite male football players using external load data. The study took place at an Italian professional football club, encompassing players from the first team, U19, and U18 teams. Players engaged in training sessions five to six times per week and participated in official matches during weekends. Players who took part in <60% of training sessions were excluded from the study to remove the subjects who had poor training continuity due to injuries or absence [18,19].

The primary objective was to assess the players’ neuromuscular readiness, weekly training loads, and the influence of these factors on their performance and efficiency on the field. Data collection occurred during every training session and match, with a comprehensive analysis conducted on a weekly basis.

This approach aimed to provide a detailed understanding of how training loads impact player neuromuscular readiness over time, thereby offering insights for optimizing training regimens and improving overall player efficiency.

### 2.2. Participants

The study involved a total of one hundred and six elite male players from the first team (n: 31; age: 24.1 ± 4.5 years; body mass: 79.7 ± 6.2 kg; height: 183.9 ± 5.3 cm), U19 (n: 44; age: 18.2 ± 0.9 years; body mass: 77.1 ± 18.6 kg; height: 182.2 ± 7.5 cm), and U18 (n: 31; age: 17.1 ± 0.7 years; body mass: 72.1 ± 6.0 kg; height: 179.1 ± 6.1 cm) teams.

Data were obtained from the club as the players were daily monitored over the course of the season. Therefore, requesting ethics committee clearance, as one would in usual research procedures, was not necessary [20]. However, all data were anonymized before the analysis, and the research was conducted following the Declaration of Helsinki to guarantee team and player confidentiality [21].

### 2.3. External Load Data Collection

External load data were collected using the WIMU Pro system (RealTrack Systems, Almería, Spain), consisting of various inertial sensors (three 3D gyroscopes with 8000°/s full-scale output range, a 3D magnetometer, a 10-Hz global positioning system, a 20-Hz ultra-wide band) whose validity and reliability have been previously tested [22,23]. The GPS devices were placed between the scapulae through a tight vest to minimize unwanted movement. All GPS devices were turned on before the 10 min warm-up to ensure an optimal signal acquisition. To avoid interunit variability, each player wore the same GPS device during the seasons.

## 3. Data Analysis

### 3.1. Calculation of the Locomotor Efficiency Index

Based on the procedure introduced in a previous study [17], LEI was calculated as the difference between the PlayerLoad^TM^ (PL) values predicted by the machine learning (ML) model and the real PL values (∆PL). The construction and selection of the ML model involved several steps, including data preprocessing, feature elimination, hyperparameter tuning, cross-validation, and model evaluation. All these steps are thoroughly detailed by Mandorino et al. [17].

Random forest regression (RF) was identified as the best ML algorithm and employed to predict players’ training/match PL [24,25] through seven external load metrics (Table 1), which were identified in the previous study [17] as the most important features to predict the target variable. A positive ∆PL was interpreted as a condition where the player was able to maximize the locomotor activity and minimize the load imposed on the body compared to the expected value predicted by the ML model. Differently, a negative ∆PL indicated that the player cumulated a higher PL than the value predicted by the model, suggesting a decrease in the player’s locomotor efficiency [17]. Considering the high individual variability [26], the ∆PL was reported using a z-score transformation (LEI), calculated for each player individually, based on data from the two entire seasons:

$$LEI=\frac{Individual\;∆PL-Individual\;∆PL\;average}{Individual\;∆PL\;standard\;deviation}$$

If the players completed two training sessions per day, the final LEI for the day was calculated as the average of the LEI values from both sessions.

### 3.2. Definition of the Different Training Scenarios

#### 3.2.1. Weekly Readiness

The players’ weekly neuromuscular readiness was individually calculated as the weekly average of the LEI values. According to the results, three different conditions were identified within each week by using the following cutoffs:

- Bad readiness: players exhibited a weekly LEI value lower than −0.5.
- Normal readiness: players exhibited a weekly LEI value between −0.5 and 0.5.
- Good readiness: players exhibited a weekly LEI value higher than 0.5.

#### 3.2.2. Week-to-Week Load Fluctuation

The weekly load was calculated as the sum of the load of all training sessions and matches over a period of one week. The weekly load was calculated for total distance, distance > 25.2 km/h (m), and mechanical load (cnt) (i.e., number of accelerations > 3.5 m/s^2^ + number of decelerations < −3.5 m/s^2^). The fluctuation in these external load parameters from week to week was quantified as a percentage change using the following formula:

$$\frac{Weekly\;Load\;(w+1)-Weekly\;Load\;(w)}{Weekly\;Load\;(w)}\times 100$$

where w + 1 represents the most recent week and w the previous week. Concerning the week-to-week fluctuation, five different conditions were arbitrarily classified for the w + 1 load compared to the w:

- Large decrease: <30% of the individual weekly load;
- Moderate decrease: between −30% and −10% of the individual weekly load;
- No variation: between −10% and +10% of the individual weekly load;
- Moderate increase; between +10% and +30% of the individual weekly load;
- Large increase: >30% of the individual weekly load.

#### 3.2.3. Week-to-Week LEI Variation

**Figure 1:**
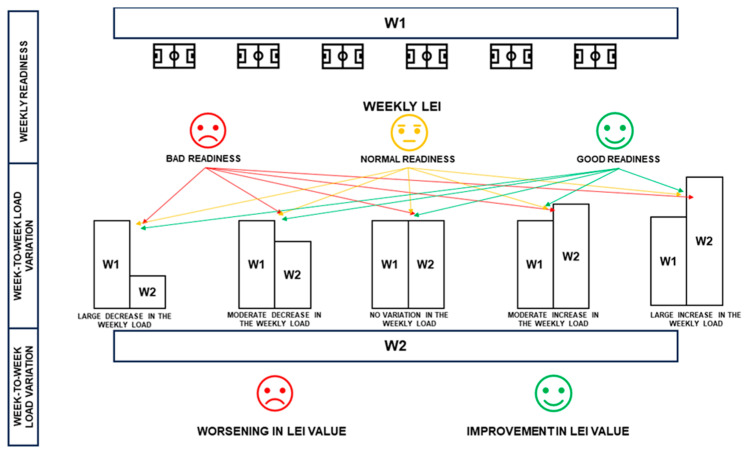
Summary of the steps performed to define the different training scenarios involved in the statistical analysis. W1 = first week; W2 = second week.

To analyze the week-to-week variation in the LEI, we calculated the absolute difference between the weekly LEIs of the most recent week and the preceding week. The steps undertaken to identify the various training scenarios are summarized in Figure 1.

## 4. Statistical Analysis

A traditional generalized linear mixed model (GLMM) was utilized to investigate the relationship between all possible two-way interactions (weekly readiness and week-to-week load fluctuation) and the week-to-week variation in LEI, which was designated as the dependent variable. To account for the repeated measurements, player identity was included as a random effect in the model. The dataset was divided into three subsets based on weekly readiness (bad, normal, good). Within each subset, a GLMM was fitted to determine the optimal week-to-week load fluctuation (large decrease, decrease, no variation, increase, large increase) that maximizes the week-to-week LEI variation. The GLMM was applied separately for each subset and for each of the selected weekly load parameters (total distance, distance > 25.2 km/h, mechanical load). The standardized regression coefficient (β) was employed to quantify the effect size of individual predictors and to identify which interaction was most significant in explaining the variation in the dependent variable [27,28]. The two-way interactions were compared with the week-to-week load fluctuation condition identified as “no variation in the weekly load”. Therefore, the analysis aimed to assess whether the other load manipulation strategies provided an advantage in improving weekly readiness compared to keeping the weekly load constant. The significance level was set at *p* < 0.05. The software used for the statistical analysis of the data was IBM’s SPSS Statistics (version 27, SPSS, Inc. Chicago, Illinois IBM Corp., Armonk, NY, USA).

## 5. Results

The mean (±SD) of the week-to-week variation in LEI is shown with respect to the different week-to-week load fluctuation conditions (Table 2).

Total Distance

**Bad Readiness Condition**

A large decrease in the weekly load resulted in a significantly higher week-to-week LEI variation (*p* < 0.01; β = 2.07).

**Normal Readiness Condition**

A large decrease in the weekly load led to a significant increase in the LEI in the subsequent week (*p* < 0.01; β = 0.82). Conversely, a moderate increase (*p* < 0.05; β = −0.40) and a large increase (*p* < 0.01; β = −0.78) in the weekly load caused a significant decrease in the LEI.

**Good Readiness Condition**

Both a large decrease in the weekly load (*p* < 0.05; β = −1.22) and a large increase (*p* < 0.05; β = −1.05) led to a significant decrease in the weekly LEI.

Distance > 25.2 km/h (m)

No significant differences were observed in week-to-week load fluctuations for distance > 25.2 km/h (m) across bad, normal, and good readiness conditions.

Mechanical Load (cnt)

**Bad Readiness Condition**

A large (*p* < 0.01; β = 1.23) and moderate decrease (*p* < 0.05; β = 0.93) in the weekly mechanical load resulted in a significant increase in the weekly LEI.

**Normal Readiness Condition**

A large decrease in the load (*p* < 0.01; β = 0.48) led to an increase in the LEI in the subsequent week. A large increase (*p* < 0.01; β = −0.48) in the weekly mechanical load had a detrimental effect on the LEI.

**Good Readiness Condition**

No significant differences were found when players were classified in the good readiness condition.

**Figure 2:**
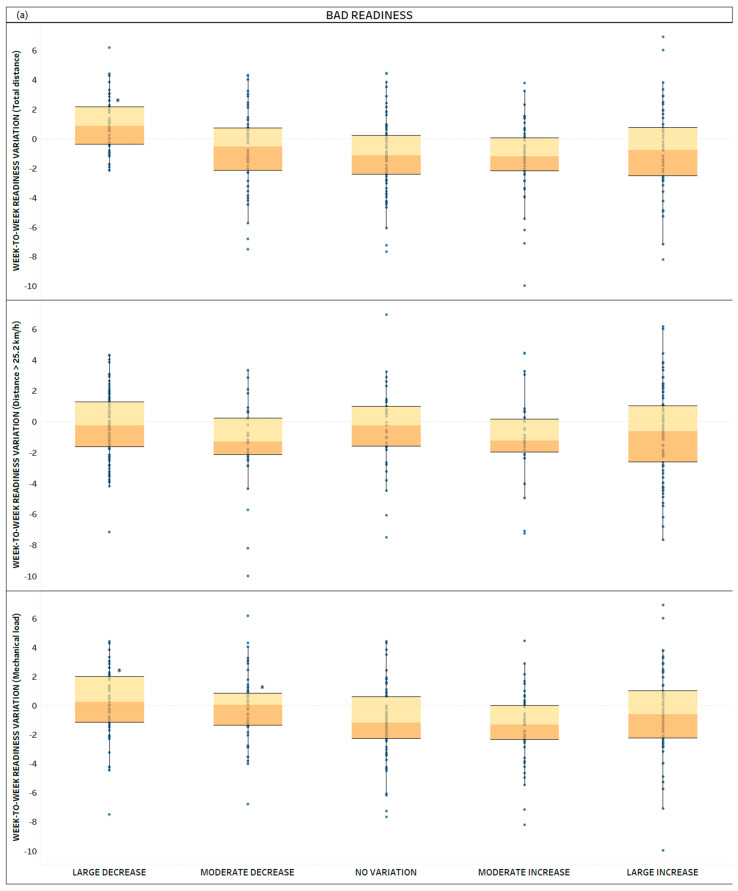

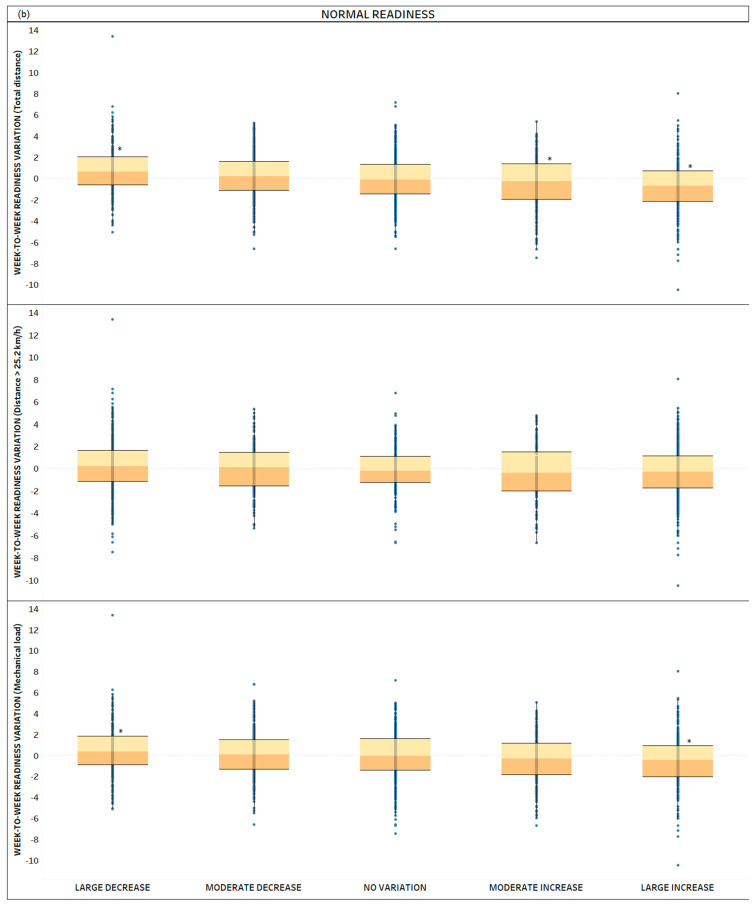

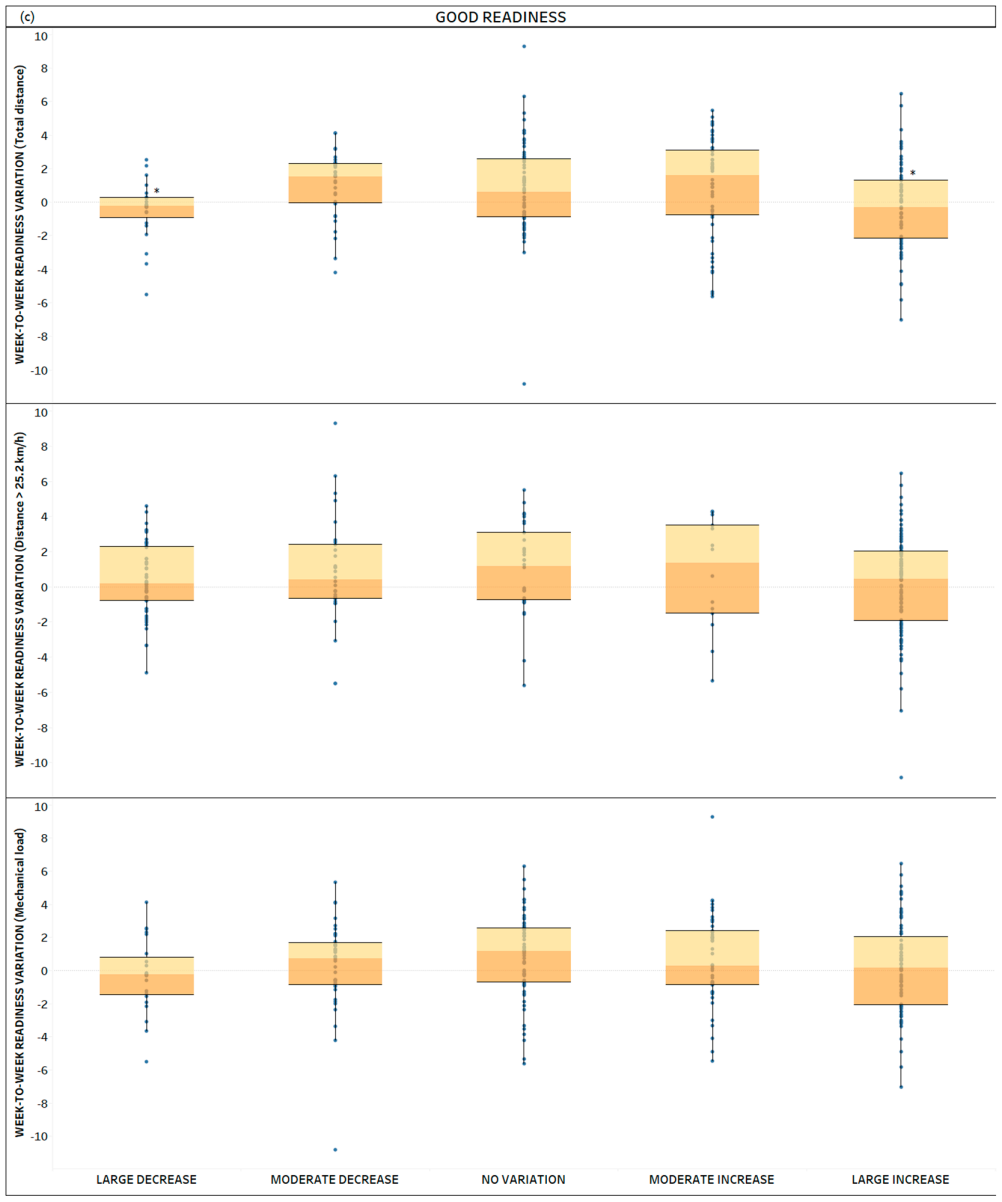
Distribution plots of the week-to-week LEI variation based on the different week-to-week load fluctuation conditions and in relation to the different readiness conditions (bad (**a**), normal (**b**), good (**c**)). * Denotes sig. difference vs. “NO VARIATION IN THE WEEKLY LOAD” condition.

All the two-way interactions for the different weekly load parameters are presented in Table 3. Box plots were used to present the median values of the week-to-week LEI variation according to the different week-to-week load fluctuation conditions (Figure 2).

## 6. Discussion

This study aimed to explore the impact of various weekly load manipulation strategies, specifically lowered, increased, or constant loads, on the neuromuscular readiness of football players, measured through the Locomotor Efficiency Index (LEI). Weekly neuromuscular readiness was determined by averaging the LEI values for each week, while the weekly load was defined as the cumulative total of all training sessions and matches, including metrics such as total distance, distance exceeding 25.2 km/h, and mechanical load. The main findings revealed that (1) the LEI is a sensitive indicator for evaluating fluctuations in players’ weekly loads concerning selected variables over the season and (2) players with low to medium readiness may benefit from reducing weekly (acute) loads, whereas significant load reductions under optimal readiness conditions could impair performance due to insufficient training stimuli.

Accurately assessing the individual workload-dose-adaptive responses to weekly training or match(es) is essential to managing players’ optimal performance. However, in football, as in other invasion team sports, these adaptive responses change more quickly because of the large number of external loads used in individual, small-group, and team drills that aim for technical, tactical, and fitness goals. This makes their assessment more difficult compared to sports with simpler training strategies. In this regard, it has been claimed that more efficient data analysis and visualization tools for coaches are needed to evaluate the effects of variations in workload volumes and intensities [8]. For this reason, Mandorino et al. [17] introduced a new locomotor efficiency index (LEI), based on a previous idea presented by Lacome et al. [29], to evaluate, for a given parameter, the effectiveness of directly comparing its predictive model with its actual measured value. By using this index, in this study, players’ weekly readiness was calculated through the difference between the PL value predicted by the ML model and the real value.

This study’s findings confirm the accuracy of the newly developed locomotor efficiency index (LEI), which predicts PL using a machine learning technique. Furthermore, it provides an answer to the question raised by Bourdon et al. [8] concerning the application of new accurate models to make use of the very large data sets generated by the current extensive daily player monitoring. Indeed, tracking the weekly fluctuations of certain selected workload variables facilitates decision making regarding a crucial choice to individualize players’ periodization: load or unload? Considering that within and between weeks, training and matches follow different dynamics, can be affected by different contextual factors, and can have variations in the number of sessions and time to prepare for the next match, detecting the week-by-week LEI variation helps coaches catch the “real-world” scenario [30].

A novel aspect of this study was the analysis of the interaction between players’ readiness and percentage of week-to-week load fluctuation in relation to total distance, distance > 25.2 km, and mechanical load. Based on the assumption that a positive or a negative LEI (i.e., positive or negative ∆PL) [17] leads to an increase or decrease in players’ locomotor efficiency [31], in our work, we identified three different conditions, such as bad, normal, and good readiness. Instead, to detect the percentage of week-to-week load fluctuation, we identified five different conditions, such as large decrease, moderate decrease, no variation, moderate increase, and large increase. The results provided in our study showed that LEI was sensitive to changes in weekly load for total distance traveled but was not affected by changes for distance traveled over 25.2 km/h. Specifically, in the bad readiness condition, the two-way interaction between weekly readiness and week-to-week load fluctuation registered a significant value for the large decrease condition (<30% of the weekly load), which indicates that a drastic reduction of the weekly load is identified as beneficial to maximize the value of LEI in the following week. This principle forms the foundation of tapering strategies, characterized by reducing the volume and/or frequency of training sessions. Beltran-Valls et al. [32] found that a two-week tapering period improved lower limb muscle power and acceleration capacities in soccer players while reducing their stress levels. Similarly, Fessi et al. [33] observed that decreasing the training load during taper weeks, by reducing the duration and frequency of training sessions but maintaining intensity, was associated with increased physical activity during matches.

These findings highlight the need to implement tapering strategies when needed in order to enhance players’ well-being. Therefore, the coaching staff plays a crucial role in optimizing the balance between training load and performance. By carefully prescribing exercises that account for players’ sustained external load and its impact on their psycho-physiological response, coaches can ensure that athletes are adequately prepared while minimizing the risk of inducing a fatigue state. Hence, the necessity of daily monitoring, thereby preserving the players’ physical capacity throughout the entire competitive season [34].

Aiming to survey the practices and perceptions of training load monitoring among coaching staff and players in elite English football, [35] demonstrated that factors such as the current match schedule, previous training, period of season, players’ fitness, and their own feelings were perceived to be “somewhat” to “very” influential in planning training. However, despite being considered speculative by the author, an intriguing finding from Weston’s study [35] is that players rated the current match schedule as more influential than coaches. As an explanation of this fact, players, in comparison to coaches, may sometimes have a more in-depth perception of the match’s demands as well as its implications for fatigue and recovery. For instance, comparing the training dose perceptions between coaches and U17/U19 professional football players during an entire season, Brink et al. [36] revealed that these young elite players perceived training as harder than coaches, which could lead to training maladaptation. Because of this, coaches may use overloading as a main training method that fits with their personal coaching philosophy. In addition, they may also find it difficult to plan weekly microcycles characterized by a workload reduction due to a potential mismatch between their perceptions and those of the players. For this reason, the findings of our work, analyzing the use of LEI in relation to different scenarios, can help coaching staff in their decision making to plan a weekly load reduction based on objective data, overwhelming the risk of a subjective mismatch with players’ perception. In every case, in our study, the beta coefficient showed how the effects of the interaction between players’ readiness and the percentage of week-to-week load fluctuation change according to the training load variation strategy, with clearer effects being registered with large variations in the training load.

According to Bannister’s fitness–fatigue model [4], performance is considered the outcome of both fitness and fatigue as positive and negative factors, respectively, which are in turn influenced to varying degrees by the exercises’ contents and order in a training session. The assumption behind this statement is that each workout elicits these two contrasting responses, which both diminish gradually over time based on their magnitude and rate. This type of model is also commonly referred to as an impulse-response model, which is based on observing the organism’s response to a perturbation in homeostasis caused by the impulse (training load) within a specific time frame. Bannister’s original model also assumes only two contrasting factors that start their after-effects at the end of the training session, which has been considered misleading by a revised version considering multiple fitness and fatigue after-effects [37]. It is also argued that while the maximal value of fatigue after-effects is taken immediately after the session, the fitness after-effects are known to be progressive, continuing from the end of the session [38]. Furthermore, this model is also considered a time-varying linear one, and to accurately estimate the relationships between training load and performance, it may necessitate a substantial number of observed units [38].

In this respect, the use of rolling averages, which have been used to determine the LEI, can help increase the accuracy of predictions. Indeed, our study bases the relationship between LEI and weekly load fluctuation on the weekly training load, which we calculate as the rolling sum of the seven previous days for the external parameters. The acute/chronic workload ratio allows for considering not only the amount of load per se but also the load that the player is ready to tolerate. Moreover, the length of the window used to calculate the acute/chronic and the presence of large spikes in workload (high fluctuation) might be important aspects to be considered in the decision-making process [39]. Our study suggests that the weekly load could also be decreased when assessing players in a “normal readiness” condition. Indeed, according to this scenario, players’ preparedness would worsen with an additional load increase. Following the general principle of progressive overload, a player should slightly exceed his load capacity in a week-to-week change in training load, as excessive or too-quick increases can provoke maladaptation and increase the risk of injury. Indeed, Piggot et al. [40] found an association between an increase in the weekly internal load (>10%) and the risk of injury in the subsequent 7 days in Australian football players. Therefore, to minimize this risk, it is suggested to limit this increase to <10% [41], which aligns with the condition of “no variation” used in our study to classify fluctuations between −10% and +10% of the individual weekly load.

Furthermore, our study revealed that when players are in an optimal state of preparedness (good readiness), it is crucial to prevent both a large decrease and a large increase in weekly load, since both were affecting a significant decrease in the weekly LEI. These findings are in line with general principles of training, which explain that low training loads do not elicit positive adaptations, while excessive loads can elicit diminished performance. A thorough understanding of the effect of training load on performance can be accomplished by considering the impact of load on the risk of injury. Based on the “work-load aetiology model” [42], workloads play a role in causing injuries through exposure, which is influenced by the overall load, and positive and negative adaptations that are governed by both the total workloads and alterations in load. Though the primary goal of coaching staff should be to determine a workload strategy that maximizes benefits while minimizing costs, both strategies of underload and overload increase the risk of injury. Excessive accumulations and significant changes in load, leading to prolonged fatigue status, have been identified as primary risk factors [43]. Conversely, an excessively reduced load can also negatively impact performance by leaving players underprepared for the demands of competition.

The final point to consider in this paper is that only variations in total distance and mechanical load showed significant associations with the neuromuscular readiness of the players. Notably, total distance registered the highest β coefficient values. McLaren et al.’s meta-analysis on team sports [44] highlights that total distance has the most robust associations with internal load and intensity variables. Similarly, a systematic review on team sports by Fox et al. [45] indicates that for most players, total distance can be used as a key indicator, along with a few others, for quantifying training load. Therefore, monitoring total distance can effectively help manage weekly training loads and implement loading or unloading strategies. This approach enables coaches and sports scientists to optimize training programs, ensuring that athletes achieve peak performance while minimizing the fatigue status.

## 7. Limitations and Future Research Perspectives

The current study has some limitations that warrant discussion. Significant associations were found only with variations in total distance and mechanical load, but not with distances exceeding 25.2 km/h. This is despite previous research indicating a strong correlation between high-intensity sprinting and fatigue-related markers [46]. The lack of association in this study may be due to methodological issues related to using percentage variations. For example, the total weekly distance consistently exceeds the distance accumulated above 25.2 km/h, resulting in a different scale of measurement. Consequently, even minor weekly fluctuations in high-speed distances can appear disproportionately large when expressed as a percentage (e.g., a reduction from 200 m to 100 m at speeds above 25.2 km/h represents a 50% reduction, yet the absolute decrease is only 100 m). Moreover, the inclusion of teams with varying training intensities and methodologies could influence the final results. Additionally, the study’s findings were limited by the lack of correlation with objective fatigue markers, which should be included in future research. Other factors, such as sleep quality and stress status, which could impact player fatigue, also need to be considered.

## 8. Practical Applications

**Figure 3:**
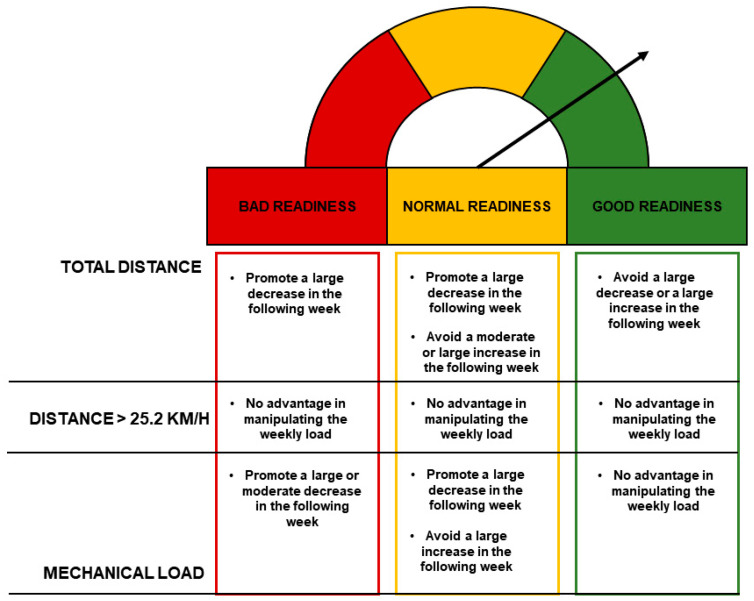
A general overview of the load management strategies to be adopted in relation to the different weekly readiness conditions.

This study provides valuable insights into the management of training loads in elite football, with implications for both coaches and players: (1) The use of the Locomotor Efficiency Index (LEI) offers a practical tool for assessing and adjusting loads to maintain or enhance player readiness and performance (Figure 3). Specifically, adjustments to the training load should be based on the weekly LEI values, reducing loads when necessary to prevent fatigue and optimize performance. (2) Given the sensitivity of the LEI to training loads, recovery protocols should be tailored to the players’ specific needs, based on their weekly LEI scores. This approach ensures that players receive appropriate recovery interventions, which are crucial during congested match periods. (3) The study highlights the benefits of non-invasive monitoring techniques. These methods are crucial for daily assessments, as they minimize the burden on players while providing reliable data to inform training decisions. By applying these practical applications, football clubs may optimize performance throughout the season, minimize the risk of injury, and maximize the physical and psychological well-being.

## 9. Conclusions

This study provides a comprehensive analysis of how varying training loads impact the neuromuscular readiness of elite football players, as measured by the Locomotor Efficiency Index (LEI). The findings confirm the LEI’s sensitivity to training load fluctuations, offering a valuable tool for optimizing player readiness and performance. The results indicate that players in lower states of readiness particularly benefit from reductions in weekly training loads, highlighting the importance of tailored training interventions to maximize performance. Moreover, inappropriate load adjustments, especially increases for players in good readiness, can negatively impact performance, underscoring the need for careful management of weekly training load.

## Figures and Tables

**Table 1:** Variables employed in the machine learning model.

Predictors	Total distance (m) Distance > 7.2 km/h (m) Number of decelerations < −2.5 (m/s^2^) Number of accelerations > 2.5 (m/s^2^) Max speed (km/h) Max decelerations (m/s^2^) Max decelerations (m/s^2^)
Target	PlayerLoad^TM^ (PL)

**Table 2:** Mean (±SD) of the week-to-week LEI variation, categorized according to the week-to-week load fluctuation conditions and further segmented based on the two distinct seasons.

Season	External Load Parameter	Week-to-Week Load Fluctuation Condition				
		Large Decrease	Moderate Decrease	No Variation	Large Increase	Moderate Increase
Season 2021/22	Total distance	0.70 (±2.33)	0.32 (±2.14)	−0.13 (±2.28)	−1.03 (±2.74)	−0.37 (±2.16)
	Distance > 25.2 km/h	0.31 (±2.35)	−0.31 (±2.28)	0.03 (±2.42)	−0.50 (±2.63)	−0.38 (±2.29)
	Mechanical load	0.30 (±2.42)	0.22 (±2.17)	−0.15 (±2.51)	−0.50 (±2.22)	−0.54 (±2.44)
Season 2022/23	Total distance	0.64 (±2.40)	−0.22 (±2.36)	−0.10 (±2.26)	−0.49 (±2.55)	−0.33 (±2.80)
	Distance > 25.2 km/h	−0.03 (±2.35)	0.01 (±2.56)	−0.07 (±1.80)	−0.38 (±2.61)	−0.27 (±2.73)
	Mechanical load	0.08 (±2.57)	−0.10 (±2.24)	−0.17 (±2.13)	−0.18 (±2.77)	−0.33 (±2.78)
Overall	Total distance	0.67 (±2.36)	0.09 (±2.25)	−0.12 (±2.27)	−0.35 (±2.66)	−0.77 (±2.50)
	Distance > 25.2 km/h	0.18 (±2.35)	−0.19 (±2.39)	−0.01 (±2.22)	−0.46 (±2.61)	−0.33 (±2.50)
	Mechanical load	0.26 (±2.48)	0.08 (±2.21)	−0.16 (±2.38)	−0.38 (±2.44)	−0.44 (±2.60)

**Table 3:** Analysis of differences in week-to-week LEI variation according to the different week-to-week load fluctuation conditions.

External Load Parameter	Readiness Condition	Week-to-Week Load Fluctuation	β	95% CI	*p*-Value
Total distance	Bad readiness	Large decrease	2.07	[1.20 to 2.94]	**0.001**
		Moderate decrease	0.41	[−0.40 to 1.24]	0.319
		Moderate increase	−0.18	[−1.03 to 0.66]	0.666
		Large increase	0.39	[−0.46 to 1.24]	0.365
		No variation	0 a	0 a	0 a
	Normal readiness	Large decrease	0.82	[0.48 to 1.17]	**0.001**
		Moderate decrease	0.23	[−0.05 to 0.52]	0.115
		Moderate increase	−0.40	[−0.73 to −0.07]	**0.016**
		Large increase	−0.78	[−1.10 to −0.46]	**0.001**
		No variation	0 a	0 a	0 a
	Good readiness	Large decrease	−1.22	[−2.43 to −0.01]	**0.047**
		Moderate decrease	0.26	[−0.84 to 1.36]	0.640
		Moderate increase	0.14	[−0.77 to 1.07]	0.753
		Large increase	−1.05	[−1.91 to −0.18]	**0.018**
		No variation	0 a	0 a	0 a
Distance > 25.2 km/h	Bad readiness	Large decrease	0.15	[−0.81 to 1.11]	0.757
		Moderate decrease	−0.94	[−2.08 to 0.20]	0.106
		Moderate increase	−0.55	[−1.77 to 0.66]	0.373
		Large increase	−0.30	[−1.27 to 0.67]	0.546
		No variation	0 a	0 a	0 a
	Normal readiness	Large decrease	0.33	[−0.02 to 0.69]	0.067
		Moderate decrease	0.13	[−0.28 to 0.55]	0.524
		Moderate increase	−0.21	[−0.66 to 0.23]	0.348
		Large increase	−0.15	[−0.50 to 0.20]	0.397
		No variation	0 a	0 a	0 a
	Good readiness	Large decrease	−0.56	[−1.70 to 0.56]	0.323
		Moderate decrease	−0.16	[−1.46 to 1.14]	0.808
		Moderate increase	−0.29	[−1.94 to 1.36]	0.728
		Large increase	−0.90	[−1.92 to 0.11]	0.081
		No variation	0 a	0 a	0 a
Mechanical load	Bad readiness	Large decrease	1.23	[0.35 to 2.12]	**0.006**
		Moderate decrease	0.93	[0.06 to 1.79]	**0.035**
		Moderate increase	−0.34	[−1.26 to 0.57]	0.459
		Large increase	0.51	[−0.33 to 1.37]	0.235
		No variation	0 a	0 a	0 a
	Normal readiness	Large decrease	0.48	[0.14 to 0.82]	**0.005**
		Moderate decrease	0.12	[−0.18 to 0.44]	0.428
		Moderate increase	−0.32	[−0.67 to 0.02]	0.062
		Large increase	−0.48	[−0.81 to −0.16]	**0.004**
		No variation	0 a	0 a	0 a
	Good readiness	Large decrease	−1.13	[−2.38 to 0.11]	0.074
		Moderate decrease	−0.56	[−1.61 to 0.48]	0.291
		Moderate increase	−0.19	[−1.20 to 0.81]	0.710
		Large increase	−0.65	[−1.54 to 0.23]	0.149
		No variation	0 a	0 a	0 a

a Reference category; β = standardized regression coefficient. CI confidence interval. Values in bold represent significant results.

## Data Availability

For the results, if possible the section number is not required. This strategy was used just to split and organize results.
